# Technological Health Intervention in Population Aging to Assist People to Work Smarter not Harder: Qualitative Study

**DOI:** 10.2196/jmir.8977

**Published:** 2018-01-04

**Authors:** Sonia Chien-I Chen

**Affiliations:** ^1^ Connected Health Innovation Centre Department of Leadership and Management Ulster University Newtownabbey United Kingdom; ^2^ Ministry of Science and Technology Taipei Taiwan

**Keywords:** health care, innovation, eHealth, technology, smart health, Taiwanese health care

## Abstract

**Background:**

Technology-based health care has been promoted as an effective tool to enable clinicians to work smarter. However, some health stakeholders believe technology will compel users to work harder by creating extra work.

**Objective:**

The objective of this study was to investigate how and why electronic health (eHealth) has been applied in Taiwan and to suggest implications that may inspire other countries facing similar challenges.

**Methods:**

A qualitative methodology was adopted to obtain insightful inputs from deeper probing. Taiwan was selected as a typical case study, given its aging population, advanced technology, and comprehensive health care system. This study investigated 38 stakeholders in the health care ecosystem through in-depth interviews and focus groups, which provides an open, flexible, and enlightening way to study complex, dynamic, and interactive situations through informal conversation or a more structured, directed discussion.

**Results:**

First, respondents indicated that the use of technology can enable seamless patient care and clinical benefits such as flexibility in time management. Second, the results suggested that a leader’s vision, authority, and management skills might influence success in health care innovation. Finally, the results implied that both internal and external organizational governance are highly relevant for implementing technology-based innovation in health care.

**Conclusions:**

This study provided Taiwanese perspectives on how to intelligently use technology to benefit health care and debated the perception that technology prevents human interaction between clinicians and patients.

## Introduction

Rapid aging makes health care studies relevant and prevalent. This study is unique as it offers qualitative perspectives on how health providers can work smarter with technology, which differs from earlier studies on technology’s ability to improve productivity through quantitative methods. This study extends the current knowledge of health care innovation by debating the perception of technology-led health care from the Taiwanese perspective.

An aging society implies a gradual decrease in a nation’s productive labor force and an increase in its dependent population. Low fertility and mortality have led to a rapidly aging population in Taiwan. Consequently, the aging population has prompted people to work harder than ever. It is widely believed that diminishing caregiver support resources for increasing elderly populations, as witnessed in most developed countries, render many current care provision models for the elderly unsustainable [1-3].

Technology-driven systems have been proposed and promoted as potential solutions to help people to work *smarter* rather than harder. Despite the application of advanced technologies, concerns surrounding information governance and Taiwan’s conservative attitude toward innovation and an actual loss of autonomy might hinder prosperous health care innovation [4]. This study aims to investigate how and why electronic health (eHealth) has developed in Taiwan and health care stakeholders’ attitudes relative to innovation in a Taiwanese context to inspire other countries encountering similar issues.

## Methods

### Overview of Methods Used

To meet the study’s aims and objectives, a qualitative methodology was adopted to obtain insightful inputs and facilitate deeper probing. Taiwan was selected as a typical case study, given its aging population, advanced technology, and comprehensive health care system. A qualitative methodology offers potential benefits that may inspire countries facing similar challenges by ensuring a better understanding of “how” and “why” eHealth has been implemented in Taiwan. This study investigated 38 stakeholders in the health care ecosystem through in-depth interviews and focus groups, which provides an open, flexible, and enlightening way to study complex, dynamic, and interactive situations through informal conversation or a more structured, directed discussion [5].

### Data Collection

The recruitment of participants was voluntary through advertisement or word-of-mouth. The participants interviewed had given verbal consent according to ethical guidelines. This study was approved by the Ulster University’s institutional review board. The participants included health care professionals, industry players, academic researchers, and government agents, and the sample was selected based on the literature review and *snow-ball* sampling. An open, flexible, and enlightening methodology facilitated the study of various complex, dynamic, and interactive situations. Interviews with participants were recorded and transcribed. The data collection, writing, and analysis progressed continuously in line with an interpretive research tradition [6]. In contrast to normal interviews, which resemble normal, direct conversations between an interviewer and interviewee [6,7], intensive interviews allow the interviewer to explore and examine a certain topic or an experience in detail; therefore, they are an effective method for interpretive inquiry. The interviewer aims to comprehend the topic while the interviewee owns the explored, pertinent experiences [8,9].

Thus, semistructured interviews that lasted up to 90 min were used to collect data. The questions included categories involving innovation and business management systems (BMS) in health care to understand the relationship between health care practice and business management. Additionally, the researchers observed the various personnel and organizational factors affecting the use of remote medical informatics to comprehend health care stakeholders’ attitudes to technology acceptance and adaptability. The semistructured interview guide comprised the following several comprehensive exploratory aspects to identify how stakeholders’ backgrounds, attitudes, competencies, and genders may relate to research outcomes:

- background information
- technology acceptance and adaptability
- levels of competency
- gender

The data transcription process followed the required reliability and validity procedures for qualitative studies.

### Data Analysis

Data were collected and analyzed until theoretical saturation was reached. The study employed a thematic content analysis [10] of a qualitative analysis with recurring themes. A thematic analysis indicated the overall consistent themes that offered an in-depth understanding of these factors. Bryman [10] states that a qualitative content analysis is “probably the most prevalent approach to the qualitative analysis of documents” and “comprises a searching-out of underlying themes in the materials being analyzed.” This was organized through deep familiarization with the data collected to further develop its major themes. These themes were then further analyzed to identify subthemes and structured to provide a comprehensive account.

According to Ruben and Babbie [11], content analysis is “essentially a coding operation,” with coding referring to “the process of transforming raw data into a standardized form.” According to Ryan and Bernard [12], a traditional content analysis “comprises techniques for reducing texts to a unit-by-variable matrix and analyzing that matrix quantitatively to test hypotheses;” the researcher can produce a matrix by applying a set of codes to a set of qualitative data assuming that the codes of interest were discovered and described beforehand.

A classical content analysis is essentially a quantitative method with a system of categories at its core and as a central tool. Consequently, the simplest type of evaluation involves counting the number of occurrences per category, assuming a relationship exists between the frequency of content and meaning. Moreover, more complex procedures can be used for the analysis of different indices that correlate two separate measurements and contingencies [13].

### Theoretical Base

This study expands the current knowledge as to how technology is applied in health care innovation using a theoretical base including both eHealth [14-16] and innovation to explore ways to help people work smarter rather than harder [17-19]. Taiwan was selected as a case study because the country has exhibited significant eHealth performance and characteristics [1-3]. This study aims to explore the relationship between technology and working smart in health care to identify meaningful research gaps.

### Definition of Work Smart

This research defines the term “work smart” as operating at a high level of efficiency and effectiveness. In contrast, the term “work hard” is a distinct and traditional labor-intensive work method that produces limited output. Technology might be smarter than manual effort, and these technological practices have purportedly replaced what has been regarded as “hard work.” The question is whether technology can help health care providers work smarter from different stakeholders’ perspectives.

### Development of Technology Application in Health Care

Although technologies have been applied in health care for decades, the outcomes remain limited. Additionally, “eHealth” refers to health services facilitated by the Internet or related technologies at the intersection of medical informatics, public health, and business [15,16]. The topic of eHealth promise and performance has been broadly discussed since the year 2000 to promote effective health care [3,20-24].

Neuhauser and Kreps [3] investigated how eHealth can improve behavioral outcomes from an eHealth application perspective and noted that the outcomes vary according to diversity in human behavior. Pagliari [24] highlighted that a gap exists between user involvement and significant impacts from eHealth adoption by investigating eHealth design and evaluation and suggested that interdisciplinary collaboration between all potential stakeholders could produce more promising outcomes. According to Kreps and Neuhauser [20], eHealth processes hold significant potential concerning the accessibility of health information for both consumers and providers; consequently, this could enhance care quality by decreasing errors, increasing collaboration, and promoting health education.

Additionally, eHealth can be applied in communications to enhance the features of interactivity, multimodality, and mass customization as well as the opportunity for users to act as producers. Although challenges remain, eHealth is worthy of research in terms of its best use to improve accessibility for vulnerable populations as well as its long-term effects on public health [25]. However, Black et al [21] argued that eHealth has more benefits in theory than in practice, and its risk management must be considered to maximize its advantages.

Therefore, more empirical applications have been adopted with the development of eHealth that encompass health information technology, eHealth record systems, and health information exchange to produce comprehensive outcomes and patient engagement [23,24,26-29]. With an aging population, more research has focused on how technology, such as telemedicine, telehealth, and telecare, can be employed to remotely control chronic conditions.

Taiwan was selected as a typical case to study eHealth implementation because of its advancements in medicine and technology [30-35], its aging population phenomenon, and health care workforce shortage. Moreover, geographic gaps between Taiwan’s rural and urban populations indicate the need for research. Therefore, the Taiwanese Government sponsored several pilot schemes to manage issues related to population aging to help health care stakeholders work smarter.

Health care stakeholders are the individuals or groups directly or indirectly affected by eHealth systems; they either suffer from the problem that the eHealth system addresses or are affected by the eHealth solution itself. These stakeholders include eHealth users, health care professionals, patients, their family members, community social workers, and researchers.

Although telecare pilot schemes in Taiwan have exhibited positive results, current literature in most western countries suggests that the application of eHealth does not always translate into working smarter [36-38]. Additionally, literature provides evidence for professional resistance to the spread of innovation in health care [39-41]. Therefore, some stakeholders would prefer to remain in the safer “hard work” arena because of the uncertainty of innovation.

A review of the literature reveals that barriers to adoption are central to the lack of interoperability standards and care provider resistance. These barriers are based on ethical and security issues, including data protection, security, primary, and confidentiality issues [29,42-45]. This study reviews literature pertaining to disruptive technology to explore the status of Taiwan’s technology adoption in health care [17].

## Results

### Key Results

**Figure 1:**
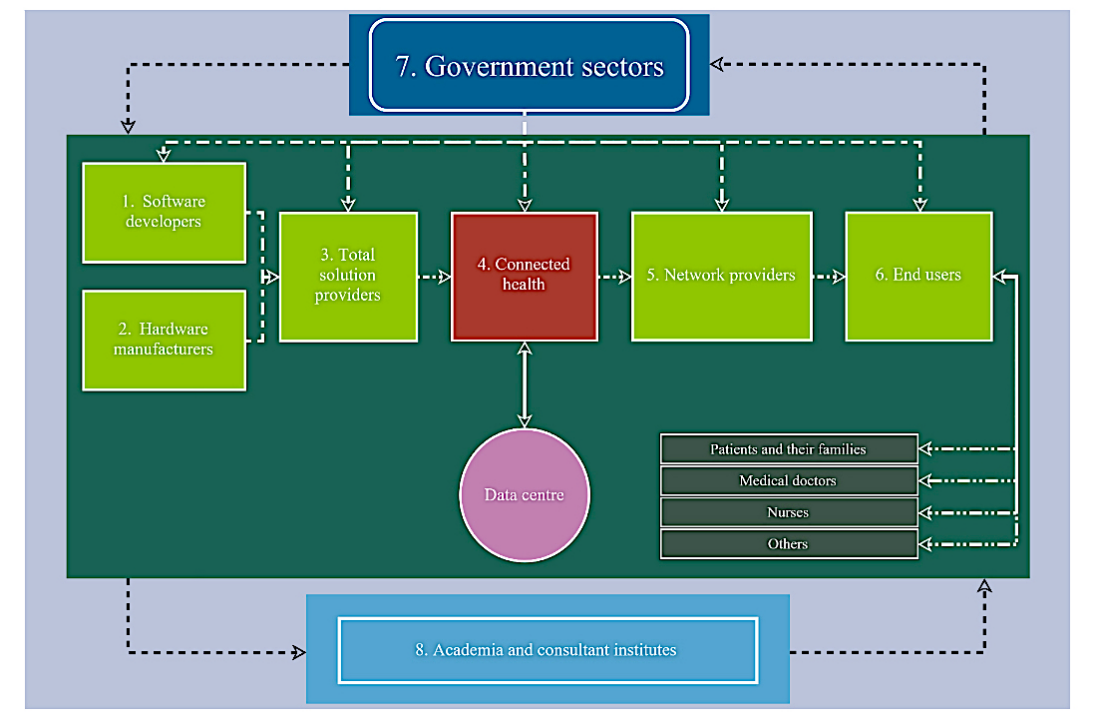
eHealth or connected health stakeholder relationship diagram.

Figure 1 illustrates the relationships and collaborations among connected health stakeholders in the business ecosystem according to the data analysis results. Interviewees were selected and grouped into 8 categories based on this business ecosystem. The 38 interviews focused on following 5 main industries: software, hardware and electronics, Internet and telecommunications, health care, and total solutions. Current regulations in Taiwan have limited the practice of eHealth for hospitals and care institutions. Although it is difficult to define such institutions as academic or industry-related, they play an important role in this field. This research collects different perspectives with the aim of inspiring more creative approaches and exploiting the advantages of health care innovation.

The interviewees were labeled according to the 8 categories shown in Figure 1. One interviewee could represent more than one category because the company might be involved in various areas in the connected health ecosystem. Therefore, 34 subcategories were labeled from number 101 to number 805, as Table 1 indicates.

Data were analyzed based on the emergent patterns to respond to the research question. As there is diversity in the qualitative analysis because of the nature of richness and complexity in the qualitative research, the purpose of the analysis was to obtain rich information from respondents. A quasistatistical analysis was conducted using N-Vivo software (QSR International) to identify key themes with the adoption of a grounded approach in this analysis. In this study, codes were derived from the data through several steps, including data cleaning, data summarizing, data analysis, and data mining. First, data were screened through the integration process to merge different terms that have the same meaning, for example, connected health could be called remote health, telehealth, and telecare in the interviews; terms were merged according to the actual meaning of the interviewees. Second, data were summarized, clustered, and categorized based on the meaning of the interviewees.

For example, some interviewees mentioned the issue of Internet connection and wireless connection, which can be summarized and categorized into technology issues or infrastructure issues depending on their context. Third, data were analyzed and extracted according to the insightful meaning of interviewees as it is the stage of the data mining process.

The interview transcriptions were coded to generate summaries for translation into key themes through N-Vivo and the author’s hand coding, as noted in Table 2. Summaries were further categorized into patient care, clinical benefits, and staff development. Additionally, leaders’ visions, personalities, authority, and management capability were extracted as leadership and management themes. Finally, the hospital or internal institution’s support system and the government or external institution’s support system or ecosystem were classified into organizational issues.

### Key Findings

The research outcomes can be classified as technology rationale, leadership management, and organizational governance, as shown in Table 2. First, respondents indicated that the use of technology could enable seamless patient care and provide clinical benefits including time management flexibility. Consequently, this bridges the gap between urban and rural access to the National Health Service. A technological learning curve could be overcome by staff development and the integration of younger and older generations. Other benefits, such as preventive medicine, have displayed decreased emergency room visit rates and provided both physical and mental total solutions to patients. One respondent stated the following:

The concept of preventive medicine through regular health recording is one of the crucial characteristics of remote health care.

101, Senior Engineer

Second, the results indicate that management’s leadership and strategic thinking are the drivers of successful health innovation. One respondent stated the following:

Although remote areas in Taiwan have limited resources compared to the urban areas, they can provide the best model for the research and development of new [telehealth] products to enter the global market.

403, Director

Finally, this investigation implies that both internal and external organizational governance are highly relevant to the implementation of technology-based innovation in health care. Government funding is vital to encourage and initiate pilot schemes to develop comprehensive business models for business sustainability.

**Table 1:** Interviewees’ categories, as summarized by the author.

Category and label number			Name
**Software developers**			
	101	Far EasTone Telecommunications	
	102	Guidercare	
**Hardware manufacturers**			
	201	Netown Corporation	
	202	Far EasTone Telecommunications	
	203	Huede	
	204	Guidercare	
	205	Acomotech	
**Total solutions providers**			
	301	Netown Corporation	
	302	Far EasTone Telecommunications	
	303	Huede	
	304	Guidercare	
	305	Acomotech	
**Connected health care service providers**			
	401	Luo Dong Care Institute	
	402	En Chu Kong Hospital	
	403	Taoyuan Fu Hsing Township Health Station	
	404	Taipei Medical University Telehealth and Telecare Center	
	405	Mennonite Christian Hospital	
	406	Taiwan University Hospital Telehealth Center	
	407	Taiwan University Hospital	
**Network providers**			
	501	Far EasTone Telecommunications	
**End users**			
	601	National Taiwan University Hospital	
	602	Mennonite Christian Hospital	
	603	Taipei Medical University TH and Telecare Center	
	604	En Chu Kong Hospital	
	605	Luo Dong Care Institute	
	606	An-Tai Hospital	
**Government sectors**			
	701	Health and Welfare Department	
	702	Sang Chung Health Center	
	703	Luo Dong Care Institute	
	704	Taoyuan Fu Hsing Township Health Station	
**Academia**			
	801	National Taiwan University’s Department of Engineering	
	802	Taipei University of Technology	
	803	Taipei University	
	804	Unified Life and Health	
	805	National Taiwan University’s Department of Medicine	

**Table 2:** Thematic output of the summarized data on eHealth/medical informatics, as summarized by the author.

Category	Summary from coding	Key themes
Patient care option	Seamless service	Technology rationale
	Infinite service	
	Connecting people	
	Preventive care	
	Personal information privacy	
	Public health education	
	Psychological and physical care	
	Quality	
	Efficacy	
Clinical benefits	Support for personnel shortages	
	Systematic and integrated patient records	
Staff development option	Cost-effective (improved medicine consumption and decreased medical costs)	
	Computer competency	
Leader’s vision	Short-sighted or insightful	Leadership and management
	Intellectual assets	
Leader’s personality	Work experience heritage	
Leader’s power or authority	Staff turnover	
	Creative capability	
	Innovation acceptance	
	Authority	
Leader’s management	Capability of execution	
	Risk management	
	Implementation management	
	Total solutions/package solution	
Government Hospital or ecosystem National health insurance	Financial	Organizational rationale
	Nonfinancial	

## Discussion

### Principal Findings

The key findings summarize the key themes into technology rationale, leadership management, and organizational governance, which emphasizes that innovation management in health care is as important as the advancement of technology itself. To sustain health care businesses, the tight collaboration among stakeholders in government, industry, and academia is significant. This section discusses the core rationales influencing health care stakeholders in adapting technology to their objectives in the interview discourse and the literature.

### Technological Rationale

Concerns with personal information security must be addressed before designing eHealth care systems; however, if the benefits can outweigh the concerns, these systems may become as popular as online banking in the future. One respondent stated the following:

I believe that someday, accessing health information will be like online banking and online shopping.

406, Head Nurse

The securing of personal medical data should not be perceived as an insurmountable problem that prevents a telecare system’s adoption and development. Technology has the aspiration to create a mutually beneficial situation that propels the following ideas: patients will obtain better health outcomes, and physicians will experience more achievements and satisfaction.

Electronic data can be easily integrated with existing hospital information systems and, ultimately, contribute by decreasing financial burdens. More systematic and integrated patient records can be developed to offer better health services and decrease diagnostic errors, ensuring quality and cost-effective benefits. One respondent stated the following:

The blood sugar levels of those who have joined the telecare project have become more controlled

402, Senior Nurse

Mechanisms to develop innovative information technology–enabled services are particularly important [46]. This research posits that the use of technologies to improve patient-centered data management has increasingly become a research area of focus [47,48].

Additionally, this research noted one significant theme: respondents described technology in health care as a crucial tool for *connecting* people rather than *replacing* them. Technology can be used to bridge gaps, specifically, the isolation between older and younger generations and also rural and urban societies. One respondent stated the following:

Technology plays a role in delivering humanity and care to people; a remote environment causes distance among people, but technology connects them.

602, MIS Director

Furthermore, clinicians were concerned with the accuracy of biometric data. The respondents addressed these concerns because they claimed that they could typically collect more accurate data than hospitals by eliminating “white coat syndrome.”

Most respondents endorsed the necessity of tele-health care systems, yet the question of how to manage them seems to be an issue in need of exploration. One respondent stated the following:

Telehealth is necessary, and how you use it will really make a difference.

403, Director

The cost of adapting technology might initially pose problems because of growth, but upgrading and renewal costs become more cost-effective in the long term. A greater acceptance of innovation is possible by objectively demonstrating its benefits and shortcomings to the public. Ultimately, an eHealth system’s benefits will outweigh any potential problems in the long term.

### Leadership Management

The respondents noted that a leader’s vision, personality, authority, and management style are highly relevant to a tele-health care project’s success.

Employees might take their innovative ideas to rival companies if their talents are not appreciated. Many cases have proven that leadership plays an important role, and respondents note that poor management causes high staff turnover and the loss of companies’ intelligent, intangible assets. One respondent stated the following:

Our previous manager did not care about innovation, but short-term profits [...] Now, our competitors have taken opportunities and have already scored some achievements by recruiting our old staff with innovative ideas…

101, Senior Engineer

Staff development activities could help some staff members absorb global trends, which might then encourage and motivate them to accept innovation. This might improve their adaptability to new technology. One respondent stated the following:

I will not consider the learning of new technology as an issue, but an opportunity for staff blending.

405, Head Nurse

Leadership attitude is a driver of eHealth success in Taiwan with collaborations among industry, government, and academia. Furthermore, literature suggests that health care lacks creativity; however, the interview findings support the assertion that leaders’ creative thinking and capability for innovation are beneficial to innovation success [49].

Many people have low innovation acceptance because of limited capability to adapt. An effective leader should be cognizant of this and properly manage the situation to further develop comprehensive staff communication accordingly. One respondent stated the following:

Any new system will have an unavoidable run-in period…You should inform people about the potential risks during the operation of a pilot scheme and have a backup plan.

403, Director

Physician support is significant because physicians have authority over patients as well as their trust. Furthermore, physician recommendations are more convincing to patients than recommendations from other sources. One respondent stated the following:

Physicians are more convincing in promoting tele-health care systems, as patients perceive them as having authority.

406, Director

### Organizational Rationale

Although an outstanding leader is important, the successful implementation of technology in health care relies on both individuals and organizations. Government funding and infrastructure expenses along with organizational support will be beneficial for both clinicians and companies when transforming innovative ideas into concrete practices. One respondent stated the following:

The government must take the lead for the first step by offering funding; otherwise, we cannot go anywhere…

101, Senior Engineer

Bureaucracy and inflexibility can be a disadvantage in these government-funded schemes. One respondent stated the following:

The public sector has this type of issue, as all projects must be reclaimed within an annual calendar period [...] but some results cannot be shown within one to two years...

403, Director

Regarding financial sustainability in health care, collaborating with unlimited needs but limited financial capability has become the norm for governments, and political calculations are often labeled as unrealistic. However, health care provisions impact everyone’s lives, and any cost-cutting plans executed by the government or employers will have a significant effect on people [50,51].

The method of integrating a resource and its ecosystem is relevant to how organizations can benefit from leadership management. Some organizations have used their knowledge, skills, and experience in exchange for assets they did not have, such as devices or networks. Moreover, the government typically has the authority to coordinate different departments and organizations. Nevertheless, a business model that considers what occurs after government sponsorship should be developed.

### Conclusions

This study dealt with the concept of “work smarter,” not work harder, by examining technology-driven systems from a qualitative perspective. It contributed to current knowledge by debating the perception that technology in health care prevents human interaction between clinicians and patients. The originality of this study lies in the focus on the relationship between technology and its relevant stakeholders in health care, which differs from the focus of recent literature that emphasizes the productivity and performance of technology. Additionally, this study extends the knowledge from technology-focused innovation to health business innovation through a qualitative lens. The Taiwanese health care system offers compelling information to support the claim that technology can be a tool to connect all stakeholders in the health care ecosystem.

The conclusion summarizes three themes for practical implications. First, the technology user should control technology through designing that serves rather than hinders. Second, leaders should manage innovation through creativity for resource integration and acquisition, and its long-term benefits should be emphasized over its short-term advantages. Third, although government funding and organizational support are relevant to health care innovation, appropriate BMS should be developed for long-term success after the government ceases its support. Interview respondents confirm that technology is beneficial when encouraging health providers to work smarter if it is used to reinforce organizations’ resources, leadership management, and collaboration with their ecosystems. To achieve this goal, the findings of this study strongly recommend health care stakeholders consider creating competitive BMS to retain business sustainability.

Although the qualitative research in a regional study can be a limitation to the research validity, it can be considered and developed in a future study. The findings emphasize how users should employ technology to enable innovation rather than constrain innovation while pursuing its novelty. Technology users should work “smarter” when using technology to add value to its adaption. Otherwise, technology will compel users to work harder by creating additional work.

## Abbreviations

BMS: business management systems
eHealth: electronic health
